# Multiallelic Rare Variants in BBS Genes Support an Oligogenic Ciliopathy in a Non-obese Juvenile-Onset Syndromic Diabetic Patient: A Case Report

**DOI:** 10.3389/fgene.2021.664963

**Published:** 2021-10-06

**Authors:** Hamza Dallali, Nadia Kheriji, Wafa Kammoun, Mehdi Mrad, Manel Soltani, Hajer Trabelsi, Walid Hamdi, Afef Bahlous, Melika Ben Ahmed, Faten Mahjoub, Henda Jamoussi, Sonia Abdelhak, Rym Kefi

**Affiliations:** ^1^Laboratory of Biomedical Genomics and Oncogenetics, Institut Pasteur in Tunis, Tunis, Tunisia; ^2^Central Laboratory of Medical Biology, Institut Pasteur in Tunis, Tunis, Tunisia; ^3^Laboratory of Transmission, Control and Immunobiology of Infections, Institut Pasteur in Tunis, Tunis, Tunisia; ^4^Research Unit on Obesity, National Institute of Nutrition and Food Technology, Tunis, Tunisia; ^5^University of Tunis El Manar, Tunis, Tunisia

**Keywords:** monogenic diabetes, whole exome sequencing, Bardet-Biedl syndrome, oligogenic inheritance, bioinformatic analysis, case report

## Abstract

Juvenile-onset diabetes may occur in the context of a rare syndromic presentation, suggesting a monogenic etiology rather than a common multifactorial diabetes. In the present study, we report the case of a young diabetic Tunisian patient presenting learning problems, speech deficits, short stature, brachydactyly, and a normal weight. Whole exome sequencing analysis revealed five heterozygous genetic variants in *BBS1, BBS4, BBS8, MKS1*, and *CEP290*. These genes are involved in the regulation of cilium biogenesis and function. We analyzed variant combinations pathogenicity using the recently developed ORVAL tool, and we hypothesized that cumulative synergetic effects of these variants could explain the syndromic phenotype observed in our patient. Therefore, our investigation suggested a genetic diagnosis of Bardet–Biedl syndrome with an oligogenic inheritance pattern rather than a monogenic diabetes. Although there is no curative therapy for this ciliopathy at the moment, a genetic diagnosis may offer other supportive care options, including the prevention of other possible clinical manifestations of this syndrome, mainly renal abnormalities, obesity, liver fibrosis, and hypertension, as well as the genetic counseling for family members.

## Introduction

Monogenic diabetes represents a rare heterogeneous group of single-gene disorders leading to functional defects of pancreatic beta cells resulting in moderate to severe hyperglycemia. These atypical diabetes forms include maturity-onset diabetes of the young (MODY), neonatal diabetes, and syndromic forms of diabetes (Antosik and Borowiec, 2016; Sanyoura et al., 2018). The disease may be inherited within families as a dominant, recessive, or non-Mendelian trait (Hattersley et al., 2018).

Monogenic forms of diabetes are often misdiagnosed as type 1 or type 2 diabetes, which has a negative outcome on the disease follow-up (Hattersley et al., 2018). Genetic diagnosis of monogenic diabetes usually improves the patient clinical care, since it enables genetic counseling for the other family members. In addition, it helps clinicians in predicting the other multisystem clinical outcomes that may be associated with the genetic defect, mostly in patients diagnosed with syndromic forms of diabetes. Indeed, these are rare multisystemic diseases requiring multidisciplinary medical care. Correct identification of such syndromes allows the anticipation, recognition, and treatment of associated complications (Sanyoura et al., 2018).

In the present study, we report the genetic characterization results of a Tunisian patient referred to Institut Pasteur in Tunis for a genetic testing of suspected MODY. Interestingly, our work reveals a genetic diagnosis of Bardet–Biedl syndrome (BBS), a multisystemic ciliopathy with various clinical features including diabetes, with an oligogenic mode of inheritance.

## Materials and Methods

### Subjects and Sample Collection

An index patient was evaluated at the National Institute of Nutrition in Tunis for management of her hyperglycemia. Then, she was referred by the treating clinician to IPT for genetic investigation of a clinical suspicion of MODY. Biochemical and immunological analyses were performed as previously described (Dallali et al., 2019), and medical records of the patient were reviewed. In addition, other available family members were also recruited after obtaining their informed consent.

### Genetic Investigation

Genomic DNA samples of the patient and her non-diabetic mother and younger sister were extracted using the FlexiGene DNA Kit (QIAGEN). Whole exome sequencing (WES) was performed for the patient using the SureSelect Human All Exon kit V6 (Agilent Technologies, CA, USA). The captured libraries were sequenced on NovaSeq 6000 System (Illumina, San Diego, CA, USA) to generate 151-bp paired-end reads.

The quality of the sequencing reads in FASTQ files was evaluated using FastQC (https://www.bioinformatics.babraham.ac.uk/projects/fastqc/), which was followed by adapter trimming using BBDuk (https://jgi.doe.gov/data-and-tools/bbtools/bb-tools-user-guide/bbduk-guide/). We align reads to the human reference genome hg19, and we subsequently call the genetic variants in a VCF file following the GATK best practices. Variant annotation was processed using ANNOVAR (Wang et al., 2010).

Prioritization of potential disease-causing variants was carried out in a set of 86 genes implicated in monogenic diabetes using Variant Annotation and Filtering Tool (VarAFT) (Desvignes et al., 2018). The gene list was prepared through a literature review using PubMed (https://www.ncbi.nlm.nih.gov/pubmed) (Supplementary Table 1). We first excluded variants with minor allele frequency (MAF) > 0.01 in gnomAD (http://gnomad.broadinstitute.org) and in GME database (http://igm.ucsd.edu/gme/). Then, we retained non-synonymous, non-sense, frameshift, and splice site variants as they are more likely to have a functional effect. Non-synonymous variants were filtered to include those predicted to be damaging by at least eight of 14 *in-silico* pathogenicity prediction software packages (Supplementary Methods). In addition, we selected variants predicted to alter splice sites by Human Splicing Finder database (Desmet et al., 2009). Finally, we assessed the coverage of the genes harboring the filtered variants using Depth and Coverage Analysis (DeCovA) (Dimassi et al., 2015).

We evaluated the prioritized genetic variants by an intensive research in bioinformatics databases including PubMed (https://www.ncbi.nlm.nih.gov/pubmed), ClinVar (https://www.ncbi.nlm.nih.gov/clinvar), VarSome (https://varsome.com/), and LOVD (https://www.lovd.nl) to determine if they were previously reported, and if so, the clinical characteristics of the carriers and the reported genotype–phenotype correlations. Residue conservation was determined using the ConSurf server (https://consurf.tau.ac.il/). In addition, we used the machine-learning tool ORVAL to predict whether combinations of digenic variants were likely to be pathogenic (https://orval.ibsquare.be/). This web platform makes the predictions using different variants, genes, and gene pair biological features, such as the CADD raw scores of the variant combinations, gene pair recessiveness, and haploinsufficiency probabilities, as well as the existence of common pathways involving gene pairs harboring the variant combinations (Renaux et al., 2019).

Besides single-nucleotide variations, we proceeded to call copy number variations (CNVs) using the R software package ExomeDepth (v1.1.10). This tool compares normalized read count data between a test and an aggregate reference made up of samples from the same sequencing run, to determine copy number at exon-level resolution (Plagnol et al., 2012). We executed ExomeDepth with the BAM file of our patient, along with an aggregate reference set of BAM files of 18 unrelated individuals, which were generated by identical bioinformatics analysis of WES raw data obtained from the same sequencing run as our index patient.

All pathogenic and likely pathogenic variants detected by the bioinformatics analysis of the WES data were subsequently validated using Sanger sequencing, as it was described in our previous work (Dallali et al., 2019).

### Structural Modeling of the Impact of the BBS Gene Mutations

The X-ray structure of the human BBSome core complex, consisting of six BBS subunits (BBS1, 4, 5, 8, 9, and 18), was retrieved from the Protein Data Bank (PDB) (https://www.rcsb.org/; PDB entry: 6XT9). Molecular visualization has been made with Dynamut2 server (http://biosig.unimelb.edu.au/dynamut2/). The analyses of the size, the hydrophobicity, as well as the intramolecular interactions involving the wild type and the mutant residues were carried out using the HOPE server (https://www3.cmbi.umcn.nl/hope/) and the DynaMut2 server.

## Results

### Case Description

**Figure 1:**
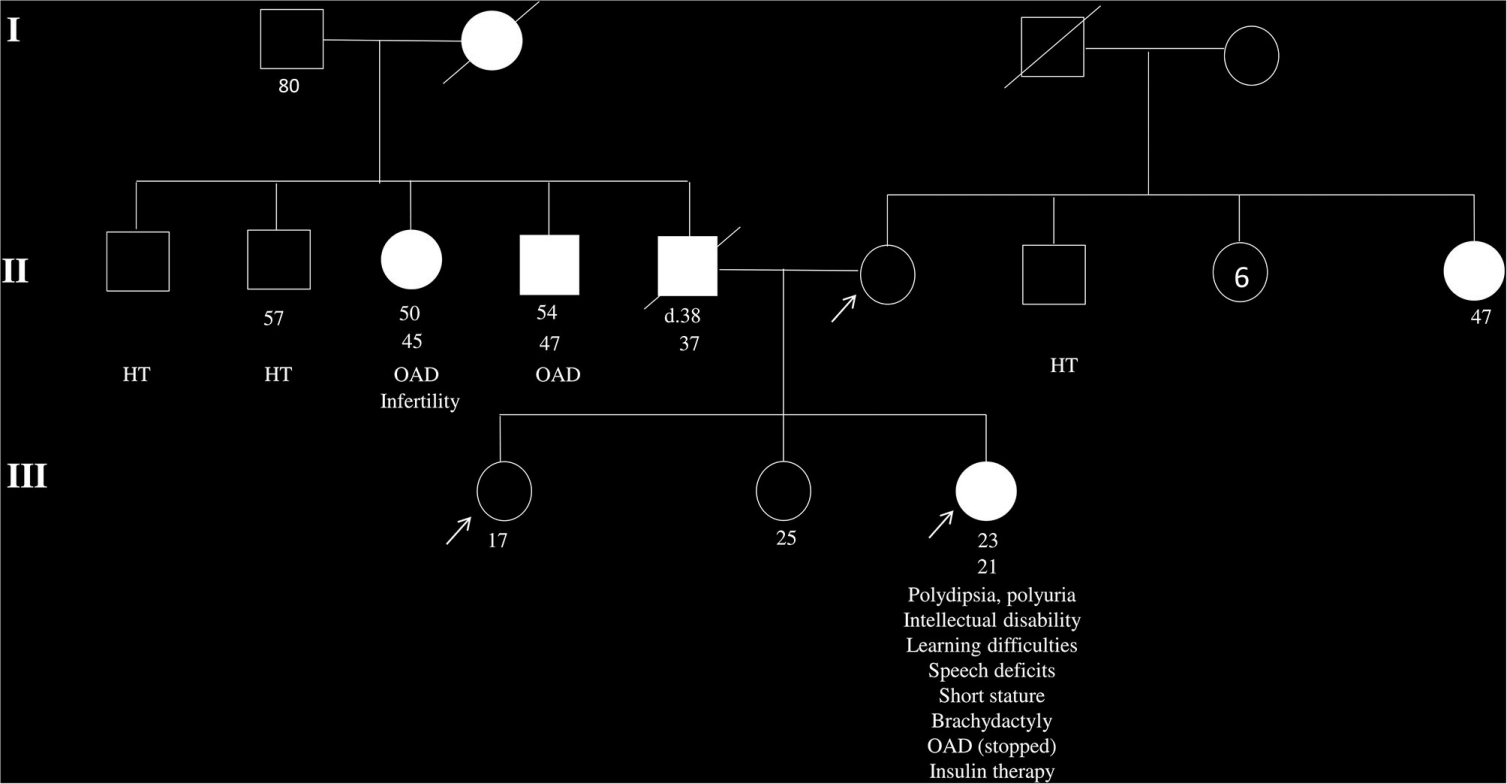
Pedigree of the family of the patient with clinical suspicion of maturity-onset diabetes of the young (MODY). The arrows indicate the patient and her non-diabetic mother and young sister, whose DNA samples are available in the present study. White squares and circles indicate healthy males and females, respectively. Black squares and circles indicate males and females with diabetes. Information below family members is ordered as follows: age at examination, age at diabetes diagnosis, clinical features, and/or specific anti-hyperglycemia treatment. OAD, oral antidiabetics; HT, hypertension.

A Tunisian girl presenting a short stature and a low BMI (17.12 kg/m^2^), born to non-consanguineous parents, has been diagnosed with hyperglycemia (FPG = 15.56 mmol/l, HbA1c = 9.3 %) at 21 years old after recurrent signs of polyuria and polydipsia. She started a healthy diet coupled with sulfonylurea uptake under the recommendation of her clinician. Besides diabetes, our patient has presented intellectual disability and post-axial brachydactyly since birth. Signs of mental impairment have remained during her childhood, with speech disorders and learning difficulties. An overview of the family history indicated the presence of diabetes in the father, two aunts, one uncle, and the paternal grandmother (Figure 1).

Fourteen months later, the clinician noticed a persistent hyperglycemia (FPG = 13.2 mmol/l, HbA1c = 8%) as well as a low level of C peptide (1.08 ng/ml). Therefore, an insulin therapy has been prescribed. Abdominal and pelvic ultrasounds were normal. Although the patient had slightly low creatinine levels (41 μmol/l), she had normal 24-h urine albuminuria (15 mg/24 h), as well as a normal estimated glomerular filtration rate (eGFR = 136.9 ml/min).

Testing for three pancreatic antibodies yielded negative results, which is not in favor of a type 1 diabetes diagnosis. Subsequently, the patient was referred to Institut Pasteur in Tunis for genetic testing of a clinical suspicion of MODY.

### Genetic Findings

The analysis of the WES performed on the index case led to the identification of two rare heterozygous non-synonymous variants in two different *BBS* genes: *BBS1* and *MKS1/BBS13*. The *BBS1* variant, c.734C > T, leads to a transition of the residue at the position 245 from proline to leucine (p.Pro245Leu). It was attributed a damaging effect by 11 *in-silico* prediction tools. The *MKS1* variant results in a missense change of arginine to cysteine at position 475 (p.Arg475Cys). It was predicted as pathogenic by 13 *in-silico* prediction software packages (Tables 1, 2). Sanger sequencing confirmed the two variants in the index patient, but revealed the presence of the *BBS1* variant (c.734C > T) in the non-diabetic mother. Furthermore, these two variants were absent in the non-diabetic younger sister (Supplementary Figure 1).

**Table 1:** List of the rare variants identified in the *BBS* genes.

**Gene**	***BBS1***	***MKS1***	***BBS4***	***TTC8***	***CEP290***
RefSeq	NM_024649	NM_017777	NM_033028	NM_198309	NM_025114
Genetic variant	c.734C > T	c.1423C > T	c.137A > G	c.889A > G	c.5237G>A
Consequence	p.Pro245Leu	p.Arg475Cys	p.Lys46Arg	p.Asn297Asp	p.Arg1746Gln
gnomAD frequency	9.19e^−05^	1^−05^	0.006	–	0.01
dbSNP ID	rs151203205	rs529604036	rs75295839	–	rs61941020
Pathogenicity score	11	13	4	3	5
Heterozygous	Patient and mother	Patient	Patient, mother, and sister	Patient and sister	Patient

**Table 2:** Pathogenicity prediction scores for the five genetic variants identified in the diabetic Tunisian patient.

**Pathogenicityprediction tools**	**Threshold score**	**BBS1-p.Pro245Leu**	**BBS4-p.Lys46Arg**	**TTC8-p.Asn297Asp**	**MKS1-p.Arg475Cys**	**CEP290-p.Arg1746Gln**
		***Prediction***	***Prediction***	***Prediction***	***Prediction***	***Prediction***
SIFT	<0.05	Deleterious	Tolerated	Tolerated	Deleterious	Tolerated
Polyphen2_HDIV	>0.453	Possibly damaging	Benign	Benign	Deleterious	Possibly damaging
Mutation assessor	>1.9	Medium	Low	Neutral	Medium	Low
FATHMM	<0	Tolerated	Tolerated	Tolerated	Tolerated	Tolerated
PROVEAN	< -2.5	Deleterious	Neutral	Neutral	Deleterious	Neutral
VEST3	>0.75	Tolerated	Tolerated	Tolerated	Deleterious	Tolerated
MetaSVM	>0	Deleterious	Tolerated	Tolerated	Deleterious	Tolerated
MetaLR	>0.5	Deleterious	Tolerated	Tolerated	Deleterious	Tolerated
CADD	>20	Deleterious	Deleterious	Tolerated	Deleterious	Deleterious
DANN	>0.8	Deleterious	Deleterious	Tolerated	Deleterious	Deleterious
UMD predictor	>50	Probably pathogenic	Polymorphism	Pathogenic	Pathogenic	Polymorphism
LRT	D, N, and U	–	Deleterious	Deleterious	Deleterious	Deleterious
Mutation taster	A, D, N, and P	Deleterious	Deleterious	Deleterious	Deleterious	Deleterious
M-CAP	D and N	Deleterious	–	Tolerated	Deleterious	–
**Harmful/total prediction tools**		**11/13**	**4/13**	**3/14**	**13/14**	**5/13**

*Pathogenicity prediction tools with threshold score or categorical classification. Reported categorical classification stand for the following: A, disease causing automatic; D, deleterious; N, neutral; P, polymorphism-automatic; U, unknown*.

Variants in these two genes are causative of BBS. Taking into account the clinical features of our patient, as well as the oligogenic inheritance pattern reported in some BBS cases (Fauser et al., 2003; Zaghloul et al., 2010), we searched for the genetic variants in the 22 *BBS* genes identified to date (Schaefer et al., 2019), independently of the pathogenicity scores for the non-synonymous variants. As a result, we found three rare heterozygous variants in *BBS4, TTC8* (tetratricopeptide repeat protein having eight domains), and *CEP290* (centrosomal protein 290) genes (Tables 1, 2). Sanger sequencing confirmed the presence of the *BBS4* variant (c.137A > G) in all three family members, which was not the case for the *TTC8* variant (c.889A > G) that was absent in the healthy mother and for the *CEP290* (c.5237G > A) variant that was present only in the index patient (Supplementary Figure 1). An in-depth analysis of the WES alignment file demonstrated a good coverage across the coding regions of the five *BBS* genes, with 93–100% of target bases covered at least 20× (Supplementary Figure 2 and Supplementary Table 2).

**Figure 2:**
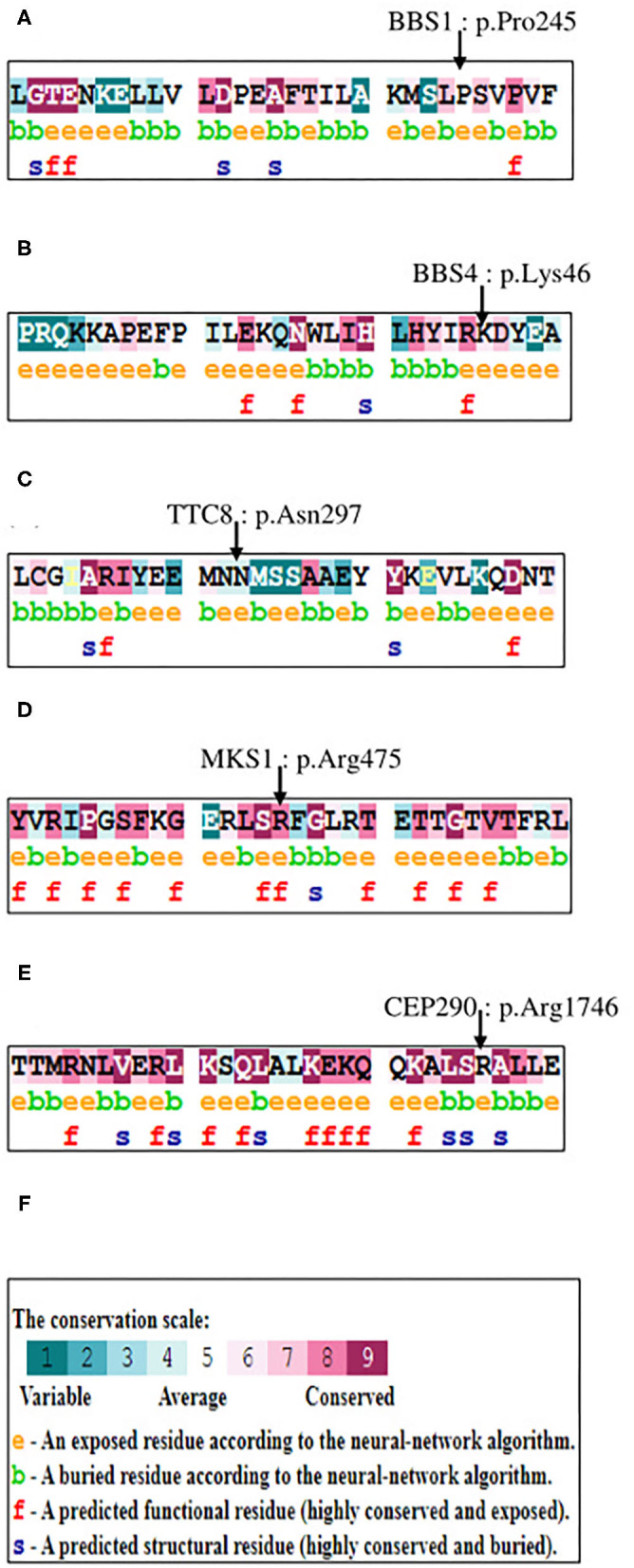
Conservation analyses results by ConSurf software. **(A)** The amino acid sequence of BBS1 (p.Pro245) is colored based on conservation scores by ConSurf. **(B)** The amino acid sequence of BBS4 (p.Lys46) is colored based on conservation scores by ConSurf. **(C)** The amino acid sequence of TTC8 (p.Asn297) is colored based on conservation scores by ConSurf. **(D)** The amino acid sequence of MKS1 (p.Arg475) is colored based on conservation scores by the ConSurf. **(E)** The amino acid sequence of CEP290 (p.Arg1746) is colored based on conservation scores by the ConSurf. **(F)** The legend of ConSurf results.

We used the ORVAL platform to explore the potential digenic or oligogenic pathogenic effects of the five rare variants. As a result, the *MKS1*-*BBS4, TTC8-MKS1, BBS1-CEP290*, and *BBS4-CEP290* variant combinations were predicted as disease-causing candidates with 95% confidence, while the *MKS1*-*CEP290* and the *BBS1*-*MKS1* variant combinations had a disease-causing probability of 99%. As shown in Figure 2, conservation analysis by ConSurf software showed high conservation scores for the BBS4 p.Lys46, MKS1 p.Arg475, and CEP290 p.Arg1746 residues that were mutated in our patient. On the other hand, the TTC8 p.Asn297 and BBS1 p.Pro245 have average conservation scores.

WES read depth analysis was performed in order to detect potential causal CNVs in the five *BBS* genes. As a result, we identified a heterozygous deletion of *CEP290* exon 5 (Supplementary Figure 3). However, Sanger sequencing did not validate this deletion, which indicates a false positive signal generated by ExomeDepth. This poor precision was reported in almost all tools developed for CNV identification from WES data (Gabrielaite et al., 2021; Gordeeva et al., 2021).

**Figure 3:**
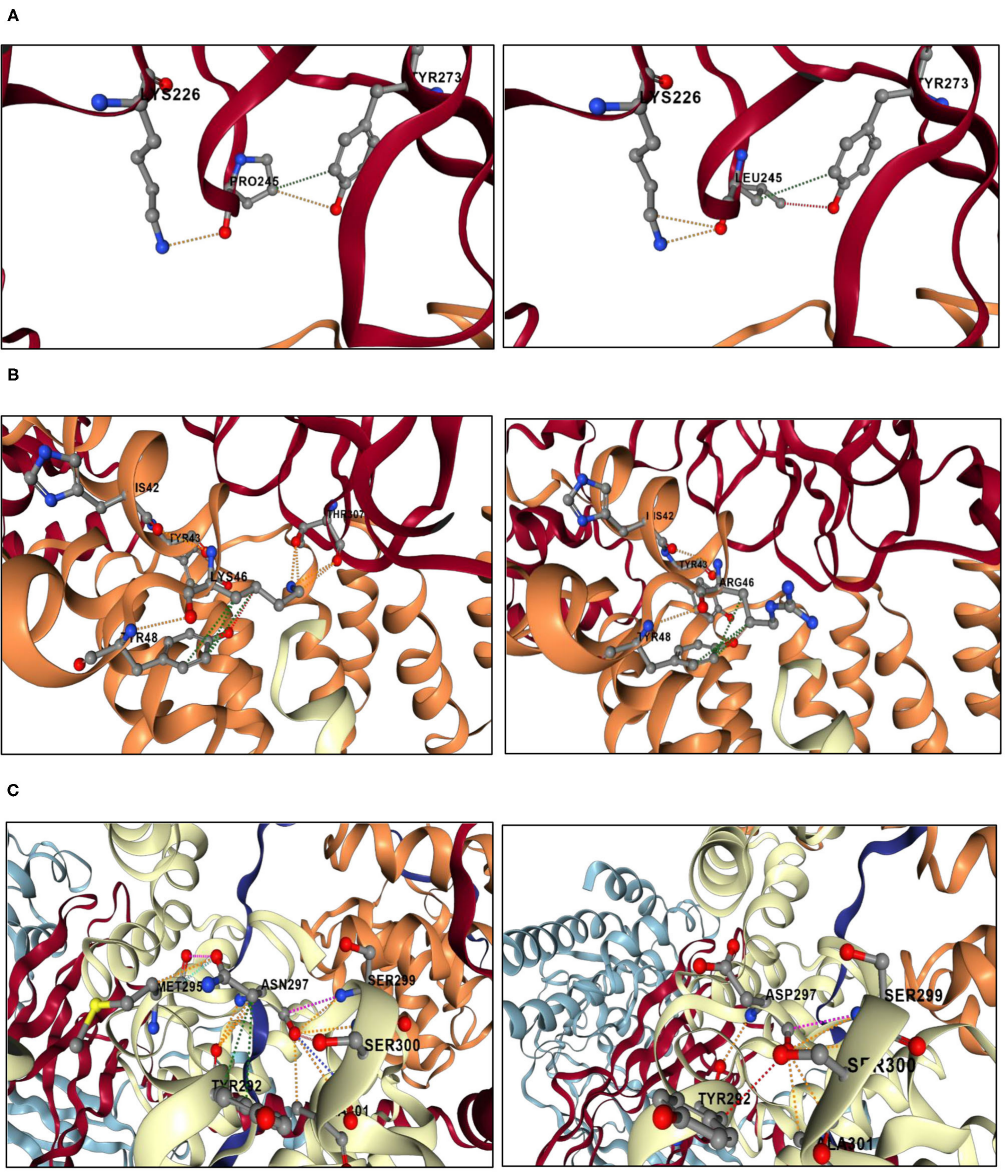
Structural analyses results of the genetic variants located in the BBSome. The analyses were performed using DynaMut2 server. **(A)** Structural analysis of the BBS1 p.Pro245Leu mutation. A fraction of BBS1 subunit is shown in red. A fraction of BBS4 is shown in orange. The left panel shows a close-up view of the interactions between Pro245 and the surrounding residues. Orange dashes represent polar bonds between Pro245 and Tyr273 and Lys226. Green dashes represent hydrophobic interaction bond between Pro245 and Tyr273. Oxygen atoms are colored in red, nitrogen atoms are colored in blue, and carbon atoms are colored in gray. The right panel shows a close-up view of the interactions between Leu245 and the surrounding residues. The mutant residue Leu245 replaces the polar bonds originally made by the wild-type residue Pro245 with the Tyr273 by weaker hydrogen bonds (shown in red dashes). **(B)** Structural analysis of the BBS4 p.Lys46Arg mutation. A fraction of BBS1 subunit is shown in red. A fraction of BBS4 is shown in orange. A fraction of TTC8 is shown in white. The left panel shows a close-up view of the interactions between Lys46 and the surrounding residues. Orange dashes represent polar bonds between Lys46, Tyr43, and Tyr48 in the BBS4 subunit, as well as between the Lys46 and the Thr308 in the BBS1 subunit. Green dashes represent hydrophobic interactions between Lys46 and Tyr48. Oxygen atoms are colored in red, nitrogen atoms are colored in blue, and carbon atoms are colored in gray. The right panel shows a close-up view of the interactions between Arg46 and the surrounding residues. The mutant residue Arg46 may not be able to make polar contacts with the residue Thr307 of the BBS1 subunit. **(C)** Structural analysis of the TTC8 p.Asn297Asp mutation. A fraction of TTC8 subunit is shown in white. The left panel shows a close-up view of the interactions between Asn297 and the surrounding residues. Orange dashes represent polar bonds between Asn297, Met295, Tyr292, Ala301, Ser300, and Ser299. Sky blue dashes represent Van der Waals bonds between Asn297 and Met295. Green dashes represent hydrophobic interactions between Asn297and Tyr292. Oxygen atoms are colored in red, nitrogen atoms are colored in blue, and carbon atoms are colored in gray. The right panel shows a close-up view of the interactions between Asp297 and the surrounding residues. The mutant residue Asp297 disrupts interactions initially made by the wild-type residue Asn297 with the residue Met295.

Structural modeling results are shown in Figure 3. BBS1, BBS4, and BBS8 are part of the BBSome, which is a heterooctameric protein complex consisting of BBS proteins 1, 2, 4, 5, 7, 8, 9, and 18.

The BBS1 mutation (p.Pro245Leu) is located in the N-terminal beta-propeller domain, which participates in the BBSome assembly *via* its binding to the BBS4 N-terminal TPR domain. The mutant residue Leu245 disrupts the polar interaction with Tyr273, which may disturb the special backbone conformation initially induced by the wild-type proline residue (Figure 3A).

The BBS4 mutation (p.Lys46Arg) is located at the interface of the BBS4 TPR domain and the BBS1 beta-propeller domain. It introduces a bigger residue that may not be able to make polar contacts with the residue Thr307 of the BBS1 beta-propeller domain, which can disturb the dimer interaction as well as the BBSome core complex assembly (Figure 3B).

The BBS8 mutation (p.Asn297Asp) is also located in a TPR domain. It leads to the substitution of the neutral asparagine into a negatively charged aspartic acid, which disrupts interactions with the residue Met295 (Figure 3C).

## Discussion

In the present study, WES analysis resulted in the identification of five heterozygous genetic variants in *BBS1, BBS4, BBS8, MKS1*, and *CEP290* genes in a young Tunisian patient referred to a genetic testing of a MODY clinical suspicion.

Variants in these five genes are causative of BBS, an emblematic ciliopathy characterized by a clinical and genetic heterogeneity. It is a multisystemic disorder with the following primary features: retinal degeneration, central obesity, postaxial polydactyly, learning problems, and renal abnormalities. Moreover, BBS is often complicated by other minor symptoms including hepatic fibrosis, diabetes mellitus, neurological problems, speech and lingual deficits, facial dysmorphism, dental anomalies, developmental delay, hypertension, brachydactyly/syndactyly, cardiovascular anomalies, reproductive anomalies, short stature, and hearing loss (Khan et al., 2016). Twenty-two *BBS* genes have been identified so far, and mutations are found in about 80% of the known BBS patients (Schaefer et al., 2019). This pleiotropic ciliopathy is mainly considered as an autosomal recessive disease. However, oligogenic inheritance has been shown in some BBS families (Katsanis, 2004; Zaghloul et al., 2010; Gazzo et al., 2016).

A clinical evaluation indicated that our patient presented some clinical features of BBS, such as mental delay, speech deficits, learning problems, brachydactyly, short stature, as well as diabetes (Table 3). Therefore, our result would suggest a BBS genetic diagnosis. Indeed, it was reported that a diagnosis of BBS should be considered in any individual with any of its major features [Bardet-Biedl Syndrome Overview—GeneReviews®—NCBI Bookshelf (2020)]. A review of literature revealed some BBS cases who do not present many major features of BBS. Cannon et al. (2008) reported two BBS brothers presenting only with a pigmentary retinopathy and postaxial polydactyly, who were homozygous positive for the presence of the most common mutation in *BBS1* (p.Met390Arg). Similarly, Pawlik et al. (2010) reported a novel homozygous *BBS12* mutation in three affected individuals in a family with postaxial polydactyly and late-onset retinal dysfunction (progressive night blindness started between 13 and 15 years of age in all the patients). The adult-affected individuals did not show any renal anomalies, obesity, intellectual disability, or hearing loss (Pawlik et al., 2010). The authors suggested that these cases serve to highlight the degree of clinical variability observed in BBS, which may be underdiagnosed in patients with milder phenotypes as observed in our index patient.

**Table 3:** Comparison of the clinical features of the patient with those of the Bardet–Biedl syndrome.

**Clinical features of the Bardet–Biedl syndrome**	**Frequency in BBS patients (%) (M'hamdi et al., 2014a)**	**Clinical features of the index patient**
**Major features**		
Rod-cone dystrophy	90–100	–
Obesity	72–92	–
Polydactyly	63–81	–
Learning problems	50–61	+
Renal anomalies	20–53	–
**Minor features**		
Speech deficits	54–81	+
Development delay	50–91	+
Diabetes mellitus	6–48	+
Dental anomalies	51	–
Congenital heart disease	7	–
Brachydactyly	46–100	+
Deafness	11–12	–

The genetic variant identified in *BBS1*, c.734C > T/p.Pro245Leu, is located in the beta-propeller domain. Besides its role in BBSome assembly, this domain interacts with ARL6, a GTPase causative for BBS3, which allows the BBSome recruitment to the membrane and the cargo recognition (Klink et al., 2020). It plays a pivotal role in promoting the biogenesis of ciliary membrane in fetal, retinal, adipose, cardiac, skeletal, and pancreatic tissues by amassing the level of GTP. The *BBS1* genetic variant detected in our patient was previously identified, along with a heterozygous *BBS9* variant, in a non-obese patient, presenting retinitis pigmentosa, postaxial polydactyly, and renal anomalies (Deveault et al., 2011). This patient did not have diabetes or intellectual disabilities diagnosed in our study case. Indeed, *BBS1* mutations were reported in a wide spectrum of phenotypes ranging from non-syndromic retinitis pigmentosa to relatively mild forms of BBS. In addition, the most common *BBS1* mutation p.Met390Arg, reported in many BBS patients, was identified in control individuals and in unaffected parents of BBS patients in some studies, which would indicate that cis—or trans—acting modifiers may influence the disease phenotype (Estrada-Cuzcano et al., 2012).

The *BBS4* variant c.137A > G/p.Lys46Arg was previously reported in two European BBS patients, participating in a triallelic inheritance with a compound heterozygous *BBS1* mutation for one patient and in a diallelic digenic inheritance with a heterozygous variant in *BBS2* for the other patient (Fauser et al., 2003; Deveault et al., 2011). BBS4 localizes around the centrosome and basal body, and it may act as an adaptor protein assisting the loading of cargo proteins for intracellular transportation within the cilium (Hirano et al., 2020). Our structural analysis demonstrated the localization of this genetic variant at the BBS4–BBS1 interface, which may disrupt the dimeric interactions between the two subunits of the BBSome. However, VarSome attributed a benign classification to the *BBS4* variant, mainly due to its occurrence at a homozygote state in seven individuals according to the gnomAD database. In addition, this variant was carried by the non-diabetic mother and younger sister, which may exclude its potential pathogenic effect.

The *TTC8* variant c.889A > G/p.Asn297Asp was not reported in genetic databases, and it disrupts some interactions within the BBSome. However, its presence in the non-diabetic younger sister may exclude a potential pathogenic effect. *TTC8* encodes for the BBS8 protein probably involved in protein–protein interactions (Khan et al., 2016). Thus far, 16 families with the *TTC8* genetic abnormality have been reported. Although full clinical information was not available for some cases, most of the cases in these families exhibit classical BBS without obvious differences in phenotypes (Sato et al., 2019).

The *MKS1* variant c.1423C > T/p.Arg475Cys was attributed a deleterious effect by almost all *in-silico* pathogenicity prediction tools and was absent from the healthy mother and younger sister, which favors its potential impact on the protein function. MKS1 is among the six MKS protein complex that functions mainly at the ciliary transition zone. It contributes in regulating entry and exit of specific membrane proteins to and from the cilium (Khan et al., 2016). Goetz et al. (2017) have demonstrated that MKS1 functionally interacts with the BBS4 subunit of the BBSome to mediate trafficking of specific trans-membrane receptors to the cilium. As for association with diseases, functional experiments have demonstrated that hypomorphic *MKS1* mutations, leading to only a reduction in the gene function, could cause BBS phenotype through epistatic effects on mutations in other *BBS* genes (Leitch et al., 2008).

*CEP290* encodes a protein, expressed in almost all tissues, that plays an important role in cell motility and division through the formation and stabilization of the primary cilium, the regulation of the entry and exit of the mitotic cell cycle process, as well as effects on centrosomal function, by acting as a central component of the ciliary diffusion barrier located at the transitional zone (Craige et al., 2010). Although the p.Arg1746Gln variant was attributed a deleterious effect by only five prediction tools (Table 2), a recent functional study suggested that this variant has a dominant negative effect on the regulatory function of CEP290. In fact, this mutation alters the cell proliferation cycle, the preservation of the ciliary diffusion barrier, and the integrity of the molecular composition in the primary cilium, which may contribute to alterations in neuroarchitecture (Kilander et al., 2018).

None of the identified heterozygous variants alone is sufficient to explain the phenotype of the patient, because none was fitting into the expected recessive inheritance model for the BBS. To obtain a better understanding of the possible oligogenic signature of BBS in our patient, we used the recently developed machine learning tool ORVAL. It outputs potential pathogenic effect for the *MKS1-CEP290* and *BBS1-MKS1* variant combinations with 99 % confidence. The high CADD scores of these variants, coupled with the dominant negative effect of the heterozygous CEP290 p.Arg1746Gln mutation, and the potentially epistatic effects of *MKS1* variants could explain the high disease-causing confidence generated by the ORVAL platform. Furthermore, combinations implicating the *MKS1* variant with the *BBS4* and *TTC8* variants and those implicating the *CEP290* variant with the *BBS1* and the *BBS4* variants were predicted as disease causing with 95% confidence. These results are reinforced by the absence of these variant combinations in the non-diabetic mother and younger sister. In addition, they are in accordance with the interactions between MKS1 and BBS4 demonstrated by Goetz et al. (2017), serving for the trafficking of specific cilium proteins. In another study, Zhang et al. (2014) demonstrated that heterozygous mutations in the BBSome components modulate the expression of CEP290-related ciliopathies. As *MKS1* and *CEP290* are associated with several ciliopathies (BBS, Meckel syndrome, and Joubert syndrome), it is likely that second-site modifier alleles interact with these two genes that modulate the expression and the severity of their associated diseases. Hence, we would suggest that the *BBS1, BBS4*, and *TTC8* variants could act as genetic modifiers by altering the expression of the phenotypes caused by the *MKS1* and *CEP290* mutations identified in the index patient.

At the molecular level, it is generally hypothesized that oligogenic diseases involve mutated proteins that are implicated in the same biological pathways or protein complexes (Renaux et al., 2019). In our case study, we identified mutations in *BBS1, BBS4*, and *TTC8*, which are part of the BBSome complex, whose function is to sort membrane proteins to primary cilia. In addition, our patient carries mutations in two genes regulating the ciliary diffusion across the transition zone, namely, *MKS1* and *CEP290*. Interestingly, the BBSome binds to the N-terminal region of CEP290 through BBS4 and co-localizes with CEP290 to the transition zone of primary cilia (Zhang et al., 2014). Therefore, any minor disturbance in the cilium biogenesis and development, caused by mutations in the ciliary genes, can collectively have an impact on the ciliary function.

As some of the patient's clinical features overlap with BBS, these results support the hypothesis that an oligogenic inheritance pattern could be a plausible model that may explain the syndromic phenotype observed in our patient. Thus, we would suggest that cumulative synergetic effects, of the identified variants in the *BBS* genes, could lead to the development of the BBS phenotype observed in the index patient through disturbing essential pathways in organelle biogenesis and maintenance as well as in cilium assembly. In particular, the *CEP290* dominant-negative effect variant and the *MKS1* variant, predicted deleterious by almost all *in-silico* tools, could be major contributors to the disease. Our finding is similar to the recent report of Costantini et al. (2021), suggesting an oligogenic inheritance model of five heterozygous variants located in genes known to be associated with diseases in an autosomal recessive manner.

A series of studies have been conducted to elucidate the role of oligogenic inheritance in BBS. Indeed, Katsanis (2004) has proposed a gradient model for BBS, transforming the phenotype from a monogenic to a quantitative trait, with the onset and severity of the disease being modulated by additional alleles. Furthermore, Zaghloul et al. (2010) suggested, through a combination of *in vivo* assays in zebrafish embryos, that a significant fraction of *BBS*-associated mutations have a dominant-negative mode of action. Additionally, they proposed that carriers of dominant-negative alleles may manifest subclinical BBS-associated phenotypes, which is the case of our patient carrying the *CEP290* mutation (Zaghloul et al., 2010).

To explain the early-adulthood development of diabetes, it was reported that disruption of *BBS* genes in zebrafish model was associated with an initial increase in progenitor beta cell proliferation, followed by a high rate of apoptosis in both basal and elevated glucose conditions (Lodh et al., 2016). Lodh (2019) suggested that the onset of diabetes in BBS patients occurs after the patients start depleting their beta cell mass, resulting from a higher apoptosis compared with proliferation rate. This explanation supports the low rate of diabetes susceptibility in BBS patients in childhood (2–3%), which is in accordance with the phenotype observed in our patient (Lodh, 2019).

In Tunisia, the genotype analysis in BBS-studied families revealed that the mutation spectrum of BBS genes involved *BBS1*–*BBS10* genes, with *BBS1, BBS2*, and *BBS8* being the most mutated genes (M'hamdi et al., 2014b). To our knowledge, this is the first Tunisian BBS patient with oligogenic inheritance involving *MKS1* and *CEP290* mutations. Our findings will serve to perform genetic counseling in family members, especially in the uncles and aunts presenting diabetes as well as their siblings. Regarding the younger sister, the absence of the *MKS1* and the *CEP290* mutations, which were included in the variant combinations predicted with high disease-causing confidence by the ORVAL tool, would suggest that she would not develop the disease.

In conclusion, we have characterized a diabetic patient, with whom the identification of genetic variants in BBS genes (*BBS1, BBS4, TTC8, CEP290*, and *MKS1)*, coupled with the presence of neurologic anomalies and brachydactyly, has resulted in a BBS genetic diagnosis with an oligogenic inheritance pattern. The syndromic diabetes in the index patient is likely due to a cumulative effect of variants in *BBS* genes affecting the cilium biogenesis and function. As these genes are associated with a wide spectrum of phenotypes, we think that the generation of animal models harboring oligogenic signatures, identified in BBS family-based studies, might help in establishing a better genotype–phenotype correlation. These results may have noticeable contributions to the field of syndromic conditions in order to understand the still unsolved cases.

## Data Availability Statement

The original contributions presented in the study are included in the article/Supplementary Material, further inquiries can be directed to the corresponding author.

## Ethics Statement

The studies involving human participants were reviewed and approved by Ethical Committee of The Institut Pasteur de Tunis Tunisia (Registration number IRB00005445 and FWA00010074). The patients/participants provided their written informed consent to participate in this study.

## Author Contributions

HD and RK contributed to conception and design of the study. HD and NK performed molecular and bioinformatic analyses. WK contributed to the ethical aspect. MBA and WH contributed to the immunological analyses. MM, MS, HT, and AB contributed to the biochemical analysis. FM and HJ contributed to the clinical analysis. HD drafted the manuscript. RK supervised all the analyses and edited the manuscript. SA reviewed the manuscript. All authors contributed to the manuscript revision and approved the submitted version.

## Funding

This work was funded by Institut Pasteur de Tunis (PCI-15) and the Tunisian Ministry of Higher Education and Scientific Research (LR16IPT05 and 18PJEC07-06).

## Conflict of Interest

The authors declare that the research was conducted in the absence of any commercial or financial relationships that could be construed as a potential conflict of interest.

## Publisher's Note

All claims expressed in this article are solely those of the authors and do not necessarily represent those of their affiliated organizations, or those of the publisher, the editors and the reviewers. Any product that may be evaluated in this article, or claim that may be made by its manufacturer, is not guaranteed or endorsed by the publisher.
