# Supplementary material for: Multiallelic Rare Variants in BBS Genes Support an Oligogenic Ciliopathy in a Non-obese Juvenile-Onset Syndromic Diabetic Patient: A Case Report
Source: Front Genet. 2021 Oct 6;12:664963. doi: 10.3389/fgene.2021.664963 (PMC8526562; doi:10.3389/fgene.2021.664963)
Supplement: Supplementary file 1 [file Data_Sheet_1.PDF]

Supplementary methods

Pathogenicity prediction

The impact of the non synonymous genetic variants was assessed by an approach similar to that developed by Pezzilli et al (Pezzilli et al. 2018). It comprises 14 pathogenicity-prediction software packages. These tools were chosen because of their performance and high classification records (Castellana, Fusilli, and Mazza 2016; Grimm et al. 2015; Salgado et al. 2016). For each missense variant, the score values obtained by these tools were binarized to 1 when the following conditions were met, or to 0 otherwise: SIFT score<0.05, PolyPhen2 HDIV>0.453, FATHMM<0, MetaLR>0.5, MetaSVM>0, DANN>0.8, VEST3>0.75, CADD>20, PROVEAN<-2.5, Mutation Assessor>1.9, UMD predictor≥75, M-CAP=D, LRT=D and Mutation Taster=A or D. LRT and Mutation Taster provide categorical classifications only. For LRT, the categories and their meanings are D for "deleterious," N for "predicted neutral," and U for "unknown"; for Mutation Taster, they are A for "disease causing automatic", D for "disease causing," N for "polymorphism," and P for "polymorphism automatic"; for M-CAP, they are D for "deleterious" and N for "neutral". Finally, a total pathogenicity score was obtained for each variant by summing all the single 14 binary scores. The genetic variants with a total pathogenicity score > 7 were filtered in for further investigation.

References

Castellana, Stefano, Caterina Fusilli, and Tommaso Mazza. 2016. "Chapter 22 A Broad Overview of Computational Methods for Predicting the Pathophysiological Effects of Non-Synonymous Variants." *Methods Mol Biol* 1415: 423–40.

Grimm, Dominik G. et al. 2015. "The Evaluation of Tools Used to Predict the Impact of Missense Variants Is Hindered by Two Types of Circularity." *Human Mutation* 36(5): 513–23. /pmc/articles/PMC4409520/?report=abstract (June 24, 2020).

Pezzilli, Serena et al. 2018. "Insights From Molecular Characterization of Adult Patients of Families With Multigenerational Diabetes." *Diabetes* 67(1): 137–45. http://www.ncbi.nlm.nih.gov/pubmed/28993341 (January 8, 2018).

Salgado, David et al. 2016. "UMD-Predictor: A High-Throughput Sequencing Compliant System for Pathogenicity Prediction of Any Human CDNA Substitution." *Human Mutation* 37(5): 439–46. https://pubmed.ncbi.nlm.nih.gov/26842889/ (July 9, 2020).

Supplementary Table 1. Monogenic diabetes genes list

Gene	Phenotype
ABCC8	MODY/Neonatal diabetes
AGPAT2	Lipodystrophy
AIRE	Autoimmune diabetes
AKT2	Lipodystrophy and insulin resistance
ALMS1	Alstrom syndrome (cone-rod dystrophy, hearing loss, obesity, diabetes mellitus, dilated cardiomyopathy, and progressive hepatic and renal dysfunction)
APPL1	MODY
ARL6	Bardet Biedl syndrome 3
BBS1	Bardet Biedl syndrome 1
BBS2	Bardet Biedl syndrome 2
BBS4	Bardet Biedl syndrome 4
BBS5	Bardet Biedl syndrome 5
BBS7	Bardet Biedl syndrome 7
BBS10	Bardet Biedl syndrome 10
BBS12	Bardet Biedl syndrome 12
BLK	MODY
BSCL2	Congenital generalised lipodystrophy, severe insulin resistance and diabetes
CEL	MODY
CEP290	Bardet Biedl syndrome 14
CISD2	Wolfram syndrome 2 (diabetes mellitus, hearing loss, optic atrophy and defective platelet aggregation)
COQ2	Neonatal diabetes
COQ9	Neonatal diabetes
CTLA4	Autoimmune diabetes
DCAF17	Woodhouse-Sakati syndrome (hypogonadism, une alopecia, diabetes mellitus, mental retardation and deafness)
DNAJC3	Juvenile-onset diabetes with central and peripheral neurodegeneration
DUT	Diabetes and bone marrow failure
DYRK1B	Diabetes and metabolic syndrome
EIF2AK3	Walcott Rallisson syndrome (neonatal or early-infancy insulin-dependent diabetes, multiple epiphyseal dysplasia and growth retardation)
EIF2S3	Syndromic neonatal diabetes and mental retardation
FOXA2	Childhood-onset diabetes, congenital hypopituitarism, cardiac malformation, and anal atresia
FOXP3	Syndromic autoimmune diabetes
GATA4	Diabetes with pancreatic agenesis and congenital heart defects
GATA6	Diabetes with pancreatic agenesis and congenital heart defects

GCK	MODY/Neonatal diabetes
GLIS3	Neonatal diabetes
HNF1A	MODY
HNF1B	MODY
HNF4A	MODY
IER3IP1	Microcephaly, epilepsy, and diabetes syndrome
IL2RA	Autoimmune diabetes
INS	MODY
INSR	Insulin resistance
ITCH	Autoimmune diabetes with facial dysmorphism
KCNJ11	MODY/Neonatal diabetes
KIAA2022	Diabetes with mental retardation
KLF11	MODY
LMNA	Lipodystrophy and insulin resistance
LPL	Lipoprotein lipase deficiency and transient neonatal diabetes
LRBA	Autoimmune diabetes
MAFA	Insulinomatosis and diabetes
MDH2	Familial diabetes
MKKS	Bardet Biedl syndrome 6
MKS1	Bardet Biedl syndrome 13
MNX1	Neonatal diabetes
MYO5A	Juvenile-onset diabetes
NEUROD1	MODY/Neonatal diabetes
NEUROG3	Neonatal diabetes
NKX2-2	Neonatal diabetes
NKX6-1	Juvenile-onset diabetes
PAX4	MODY
PAX6	Juvenile-onset diabetes
PCBD1	Diabetes and hyperphenylalaninaemia
PDX1	MODY/Neonatal diabetes
PIK3R1	SHORT syndrome (short stature, eye anomalies, characteristic facial features, lipodystrophy, hernias, hyperextensibility, and delayed dentition)
PLIN1	Lipodystrophy and insulin resistance
POLD1	Deafness and lipodystrophy syndrome
PPARG	Lipodystrophy and insulin resistance
PPP1R15B	Juvenile-onset diabetes with microcephaly, epilepsy and intellectual disability
PTF1A	Neonatal diabetes with cerebellar and pancreatic agenesis
RFX6	Neonatal diabetes with pancreatic hypoplasia, intestinal atresia, and gallbladder aplasia or hypoplasia

SDCCAG8	Bardet Biedl syndrome 16
SIRT1	Autoimmune diabetes
SLC19A2	Thiamine responsive megaloblastic anaemia, diabetes and deafness syndrome
SLC29A3	Pigmented hypertrichosis with insulin dependent diabetes mellitus syndrome
SLC2A2	Neonatal diabetes
STAT1	Autoimmune diabetes
STAT3	Neonatal autoimmune diabetes
TNFAIP3	Autoimmune diabetes
TRMT10A	Juvenile-onset diabetes with microcephaly, epilepsy and intellectual disability
TRIM32	Bardet Biedl syndrome 11
TTC8	Bardet Biedl syndrome 8
WDPCP	Bardet Biedl syndrome 15
WFS1	Wolfram syndrome (Diabetes mellitus, hearing Loss and optic atrophy)
ZBTB20	Primrose syndrome (macrocephaly, tall stature, intellectual disability, autistic traits, and insulin-resistant diabetes)
ZFP57	Neonatal diabetes

Supplementary table2. Sequencing coverage of each exon of the five *BBS* genes, reported as the minimum, the mean and the % of the exon with >20x coverage

Chromosome	Start position	End position	Coding exon	Minimum (x)	Mean (x)	%>=20x
***MKS1*: NM_017777**						**99.66 %**
17	56283439	56283531	exon 18	48	77.9	100 %
17	56283643	56283741	exon 17	191	204.4	100 %
17	56283825	56283908	exon 16	143	179.4	100 %
17	56284445	56284579	exon 15	16	52.9	94 %
17	56285254	56285362	exon 14	28	63.3	100 %
17	56285465	56285535	exon 13	87	120.3	100 %
17	56285873	56285944	exon 12	85	90	100 %
17	56288019	56288085	exon 11	60	72.2	100 %
17	56288340	56288383	exon 10	152	171.3	100 %
17	56289738	56289795	exon 9	129	138.2	100 %
17	56290342	56290451	exon 8	43	66	100 %
17	56291125	56291230	exon 7	55	82.4	100 %
17	56291619	56291748	exon 6	25	45.3	100 %
17	56292101	56292199	exon 5	60	85.9	100 %
17	56293448	56293604	exon 4	58	82.6	100 %
17	56294026	56294097	exon 3	104	108.4	100 %
17	56295980	56296090	exon 2	89	128.8	100 %
17	56296511	56296591	exon 1	119	158.2	100 %
***BBS1* : NM_024649**						**100 %**
11	66278130	66278177	exon 1	80	97.1	100 %
11	66278483	66278560	exon 2	94	100.9	100 %
11	66278675	66278710	exon 3	73	77.4	100 %
11	66281876	66282149	exon 4	83	166.1	100 %
11	66283010	66283057	exon 5	34	49.7	100 %
11	66283163	66283202	exon 6	64	74.4	100 %
11	66283331	66283404	exon 7	57	75.9	100 %
11	66287087	66287219	exon 8	65	107.5	100 %
11	66288740	66288847	exon 9	63	101.8	100 %
11	66290926	66291047	exon 10	106	166.5	100 %
11	66291194	66291353	exon 11	61	130.6	100 %
11	66293593	66293663	exon 12	147	199	100 %
11	66294119	66294278	exon 13	70	150.6	100 %
11	66297289	66297423	exon 14	89	103.1	100 %
11	66298364	66298499	exon 15	88	151	100 %
11	66299126	66299213	exon 16	153	160.8	100 %
11	66299421	66299508	exon 17	36	41.4	100 %
***CEP290* : NM_025114**						**93.10 %**
12	88442960	88443191	exon 54	6	23	64.10 %
12	88444130	88444210	exon 53	33	39.1	100 %
12	88447428	88447523	exon 52	55	63.1	100 %

12	88448116	88448190	exon 51	57	68.3	100 %
12	88449352	88449494	exon 50	24	46	100 %
12	88452624	88452797	exon 49	28	90.1	100 %
12	88453674	88453797	exon 48	29	41.6	100 %
12	88454606	88454771	exon 47	19	37.5	99.40 %
12	88456468	88456555	exon 46	62	85	100 %
12	88457757	88457892	exon 45	31	41	100 %
12	88462298	88462422	exon 44	22	46.4	100 %
12	88465070	88465226	exon 43	27	62.9	100 %
12	88465557	88465703	exon 42	43	81.2	100 %
12	88470998	88471121	exon 41	36	73.8	100 %
12	88471473	88471695	exon 40	13	29.5	82 %
12	88472868	88473006	exon 39	43	78.2	100 %
12	88473958	88474172	exon 38	17	49.9	89.30 %
12	88476807	88477007	exon 37	26	69.6	100 %
12	88477623	88477731	exon 36	28	38.4	100 %
12	88478362	88478629	exon 35	11	46.1	88.40 %
12	88479815	88479950	exon 34	36	42.9	100 %
12	88480167	88480275	exon 33	35	63.7	100 %
12	88481556	88481721	exon 32	15	40.2	96.40 %
12	88482808	88483264	exon 31	8	61.1	90.10 %
12	88484504	88484616	exon 30	30	53.1	100 %
12	88486457	88486609	exon 29	50	72.7	100 %
12	88487546	88487752	exon 28	26	51.8	100 %
12	88490664	88490776	exon 27	33	45.3	100 %
12	88496614	88496788	exon 26	24	41.7	100 %
12	88500451	88500682	exon 25	73	96.5	100 %
12	88500772	88500875	exon 24	16	32.4	94.20 %
12	88502842	88502958	exon 23	10	12.4	0 %
12	88504978	88505128	exon 22	32	68.9	100 %
12	88505470	88505635	exon 21	33	52.4	100 %
12	88508196	88508339	exon 20	24	48.3	100 %
12	88508874	88508959	exon 19	27	34.6	100 %
12	88510809	88510922	exon 18	28	36.6	100 %
12	88512259	88512347	exon 17	25	28.5	100 %
12	88512419	88512520	exon 16	20	33.4	100 %
12	88513890	88514053	exon 15	4	20.4	55.80 %
12	88514773	88514943	exon 14	24	41.7	100 %
12	88519022	88519146	exon 13	14	27.4	91.90 %
12	88520092	88520215	exon 12	8	24.2	67.50 %
12	88522722	88522812	exon 11	23	35.1	100 %
12	88523470	88523653	exon 10	19	32.8	99.50 %
12	88524044	88524197	exon 9	48	64.3	100 %
12	88524321	88524342	exon 8	21	24.9	100 %
12	88524941	88524995	exon 7	17	22.2	87 %

12	88530419	88530563	exon 6	25	40.8	100 %
12	88532921	88532968	exon 5	6	10.1	0 %
12	88533271	88533341	exon 4	18	21.4	98.60 %
12	88534732	88534810	exon 3	21	31	100 %
12	88534982	88535084	exon 2	20	26.9	100 %
***BBS4* : NM_033028**						**99.2 %**
15	72978568	72978592	exon 1	57	60.9	100 %
15	72987517	72987569	exon 2	78	85.5	100 %
15	73002040	73002120	exon 3	43	55.1	100 %
15	73004584	73004648	exon 4	72	90.5	100 %
15	73007631	73007743	exon 5	32	39.6	100 %
15	73009118	73009191	exon 6	18	20.7	78.10 %
15	73015134	73015188	exon 7	79	104.4	100 %
15	73016868	73016996	exon 8	61	81.5	100 %
15	73020280	73020335	exon 9	67	77.4	100 %
15	73021956	73022025	exon 10	48	64.2	100 %
15	73023645	73023798	exon 11	26	64.3	100 %
15	73023895	73024067	exon 12	71	98.4	100 %
15	73027453	73027523	exon 13	43	45.4	100 %
15	73028165	73028307	exon 14	47	51.5	100 %
15	73029102	73029304	exon 15	52	94.5	100 %
15	73029818	73029928	exon 16	59	76.5	100 %
***TTC8* : NM_198309**						**100 %**
14	89291051	89291165	exon 2	29	41.1	100 %
14	89305795	89305916	exon 3	42	63.4	100 %
14	89307208	89307272	exon 4	78	92.3	100 %
14	89307380	89307540	exon 5	56	75.4	100 %
14	89307768	89307858	exon 6	25	30.3	100 %
14	89310149	89310194	exon 7	33	40.3	100 %
14	89319314	89319400	exon 8	30	37	100 %
14	89323533	89323621	exon 9	35	39.2	100 %
14	89327565	89327676	exon 10	31	39.8	100 %
14	89336402	89336542	exon 11	55	91.9	100 %
14	89337892	89338067	exon 12	45	69.1	100 %
14	89338673	89338796	exon 13	35	62	100 %
14	89341369	89341453	exon 14	57	71	100 %
14	89343637	89343754	exon 15	67	90.9	100 %

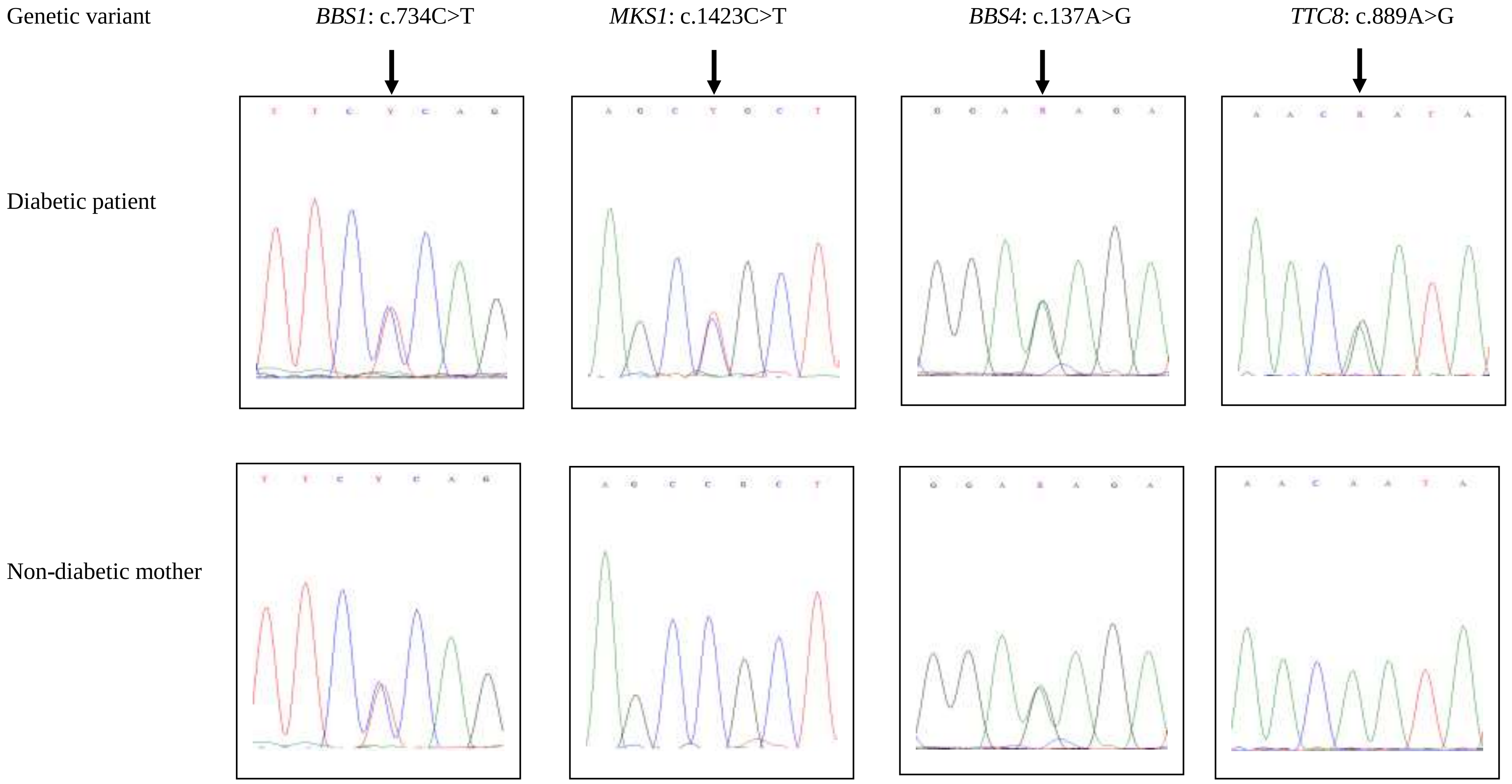
Genetic variant
BBS1: c.734C>T
MKS1: c.1423C>T
BBS4: c.137A>G
TTC8: c.889A>G
Diabetic patient
Non-diabetic mother

Non-diabetic young sister

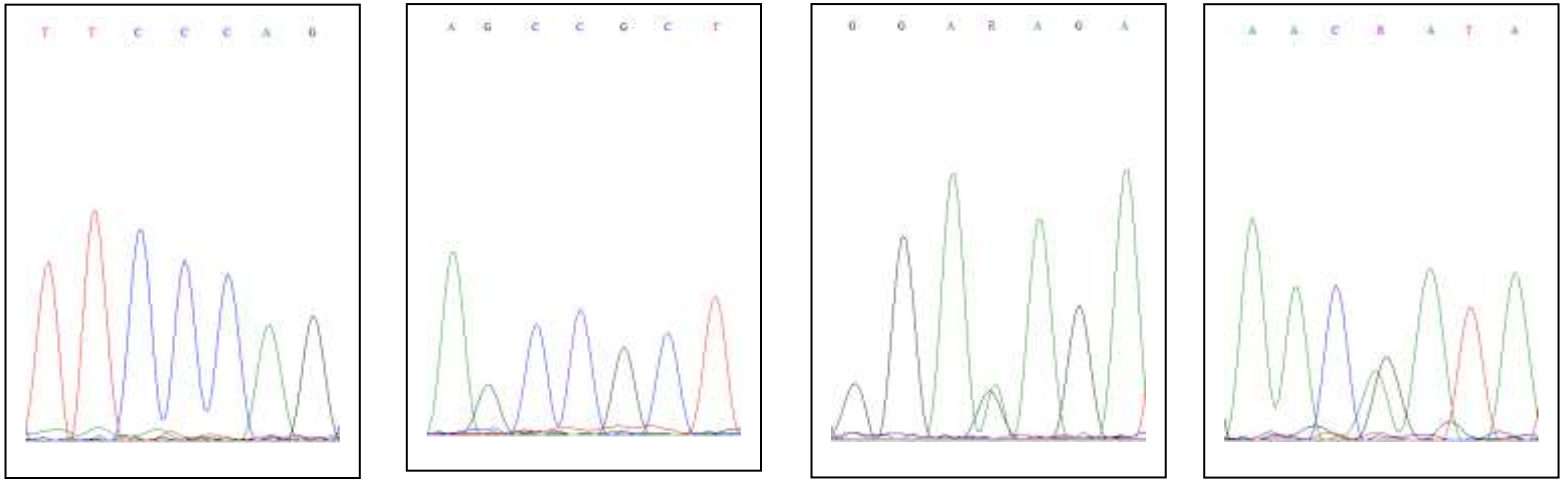

Supplementary Figure 1. Sanger sequencing results of the rare *BBS* variants in the diabetic patient and the non-diabetic mother and young sister

A)

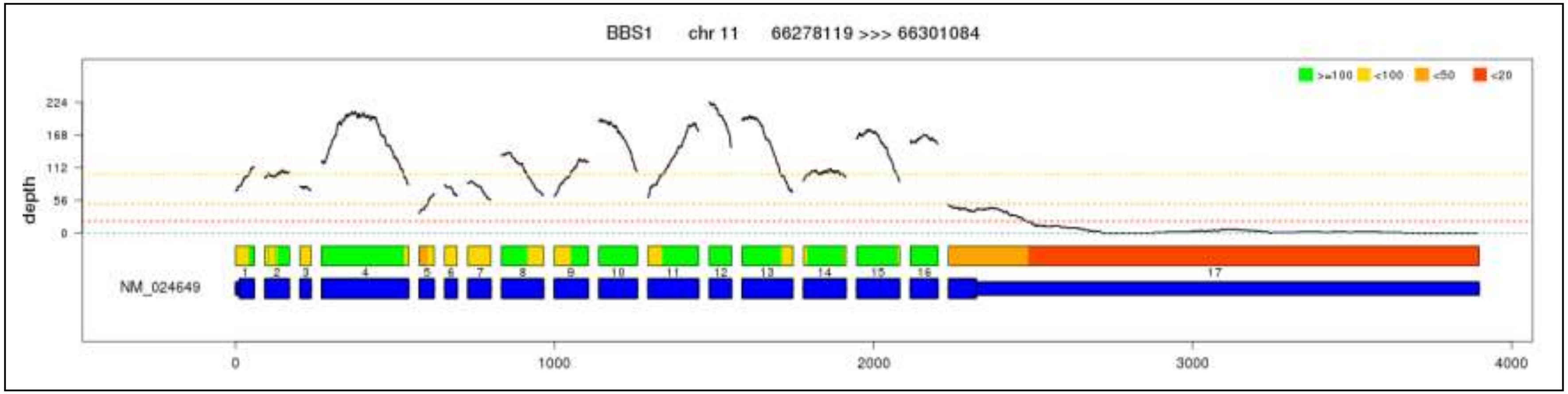
BBS1 chr 11 66278119 >>> 66301084
depth
NM_024649
>=100 <100 <50 <20

B)

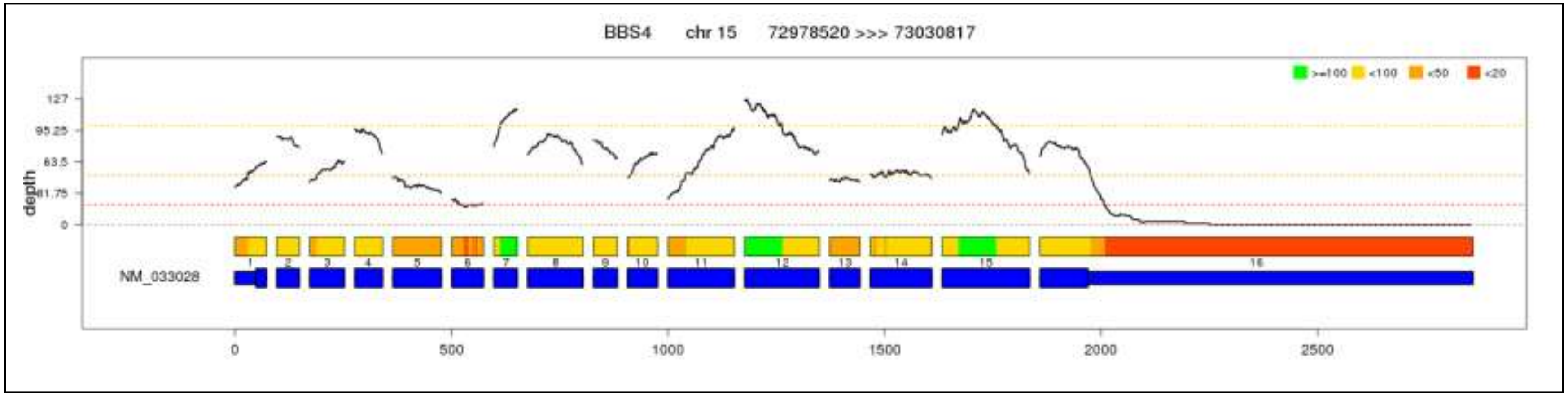
BBS4 chr 15 72978520 >>> 73030817
depth
NM_033028
>=100 <100 <50 <20

C)

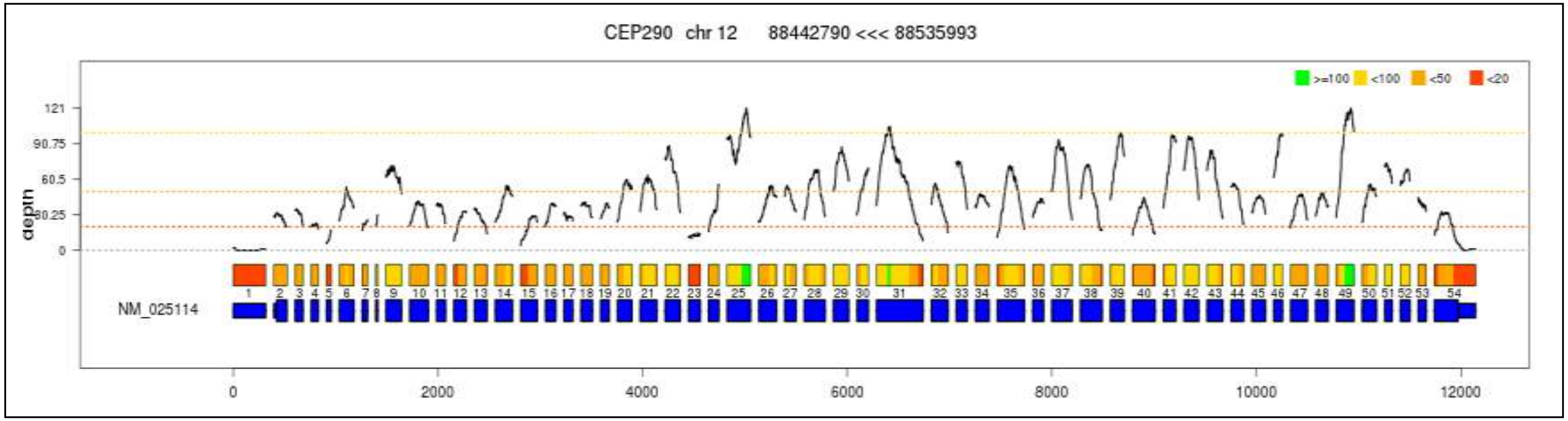
CEP290 chr 12 88442790 <<< 88535993
>=100 <100 <50 <20
depth
NM_025114
0 2000 4000 6000 8000 10000 12000

D)

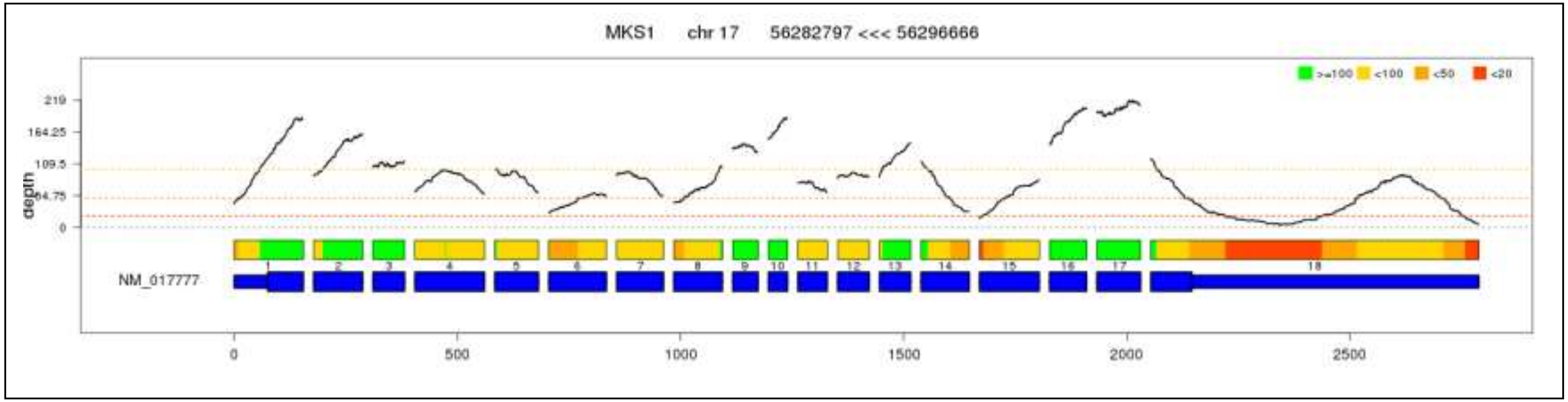
MKS1 chr 17 56282797 <<< 56296666
>=100 <100 <50 <20
depth
NM_017777
0 500 1000 1500 2000 2500

E)

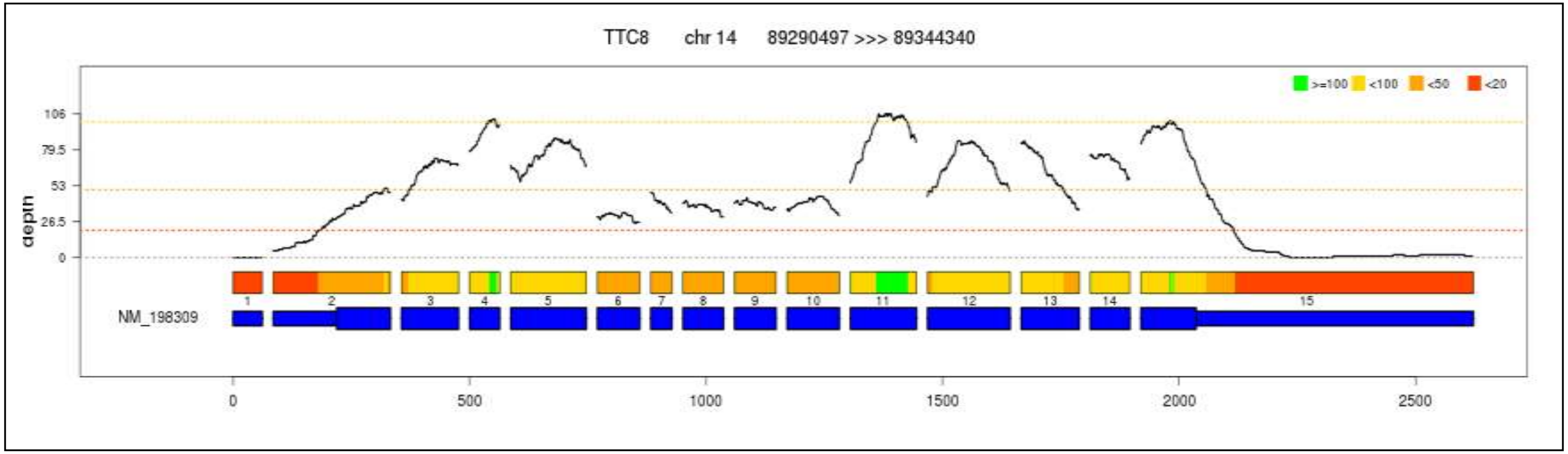

Supplementary figure 2. Coverage graphs generated by DeCovA for the five *BBS* genes harbouring prioritized variants. The read depth is showed with a black solid line, above the target regions, depicted with a colour code: green is for regions covered > 100x, yellow is for regions covered between 50 to 100x, orange is for regions covered between 20 to 50x and red is for regions covered below 20x. Below are the exons, in blue, with coding regions widened. A) Coverage graph for *BBS1* gene; B) Coverage graph for *BBS4* gene; C) Coverage graph for *CEP290* gene; D) Coverage graph for *MKS1* gene; E) Coverage graph for *TTC8* gene.

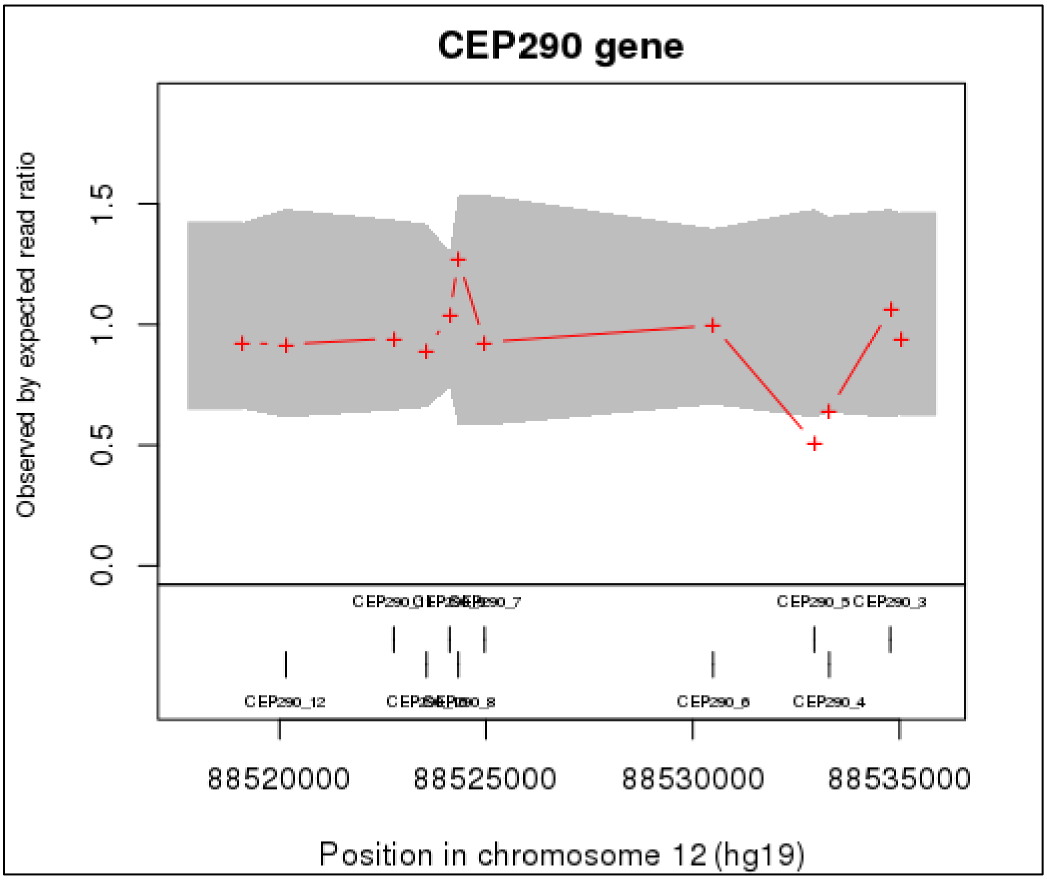

Supplementary figure 3. Heterozygous deletion of exon 5 of the *CEP290* gene identified by ExomeDepth in the patient’s exome sequence data. The red crosses show the ratio of observed/expected number of reads for the test sample. The grey shaded region shows the estimated 99 % confidence interval for this observed ratio in the absence of CNV call. As *CEP290* gene contains 54 exons, the figure shows only the first 13 exons of the gene in order to have a better resolution.
